# Success Rate and Complications of the Supraclavicular Approach for Central Venous Access: A Systematic Review

**DOI:** 10.7759/cureus.23781

**Published:** 2022-04-03

**Authors:** Atif Nazir, Khurram Niazi, Syed Muhammad Jawad Zaidi, Muhammad Ali, Saeed Maqsood, Jahanzeb Malik, Mehwish Kaneez, Amin Mehmoodi

**Affiliations:** 1 Cardiology, Rawalpindi Institute of Cardiology, Rawalpindi, PAK; 2 Internal Medicine, Rawalpindi Medical University, Rawalpindi, PAK; 3 Cardiology, Bannu Medical College, Bannu, PAK; 4 Cardiology, Mega Medical Complex, Rawalpindi, PAK; 5 Internal Medicine, Ibn e Sina Hospital, Kabul, AFG

**Keywords:** subclavian vein, critical care, central venous pressure, central venous line, landmark technique

## Abstract

Central venous catheterization plays a key role in patients that require immediate resuscitation, long-term fluid management, and invasive monitoring. The supraclavicular (SC) and infraclavicular (IC) approaches are utilized for central venous catheterization and both have their benefits and limitations. In this systematic review, we aim to explore the success rate and various complications of the SC technique.

A literature review was conducted on the PubMed, EMBASE, Scopus, CINAHL, and Cochrane databases. All relevant original articles that evaluated success rates and complications of SC access were retrieved and included for qualitative synthesis.

After screening 1040 articles, 28 studies were included for further analysis. The overall success rate of SC access ranged between 79% and 100%. The overall complication rate in SC access ranged between 0% and 24.24% (Mean: 4.27%). The most prevalent complication was arterial puncture (1.39%) followed by catheter malposition (0.42%).

The SC approach can be used as an alternative to the IC technique because of its low access time and high success rate. The SC approach should be more commonly used in day-to-day central venous cannulation. Further studies on the role of ultrasound guidance are warranted for the SC approach.

## Introduction and background

Central venous catheterization is a necessary procedure in patients requiring hemodialysis, volume resuscitation, central venous pressure monitoring, the administration of inotropes, temporary transvenous pacing, and long-term chemotherapy [1]. There are three important venous routes for central venous access: (i) internal jugular; (ii) femoral; and (iii) subclavian. Each one has its advantages, disadvantages, and potential complications [2-3]. Subclavian vein (SCV) catheterization has several anatomic advantages, including large diameter, good patency rate, constant anatomical position, and absence of venous valves. SCV catheterization also carries a lower risk of catheter-mediated infection when compared with internal jugular or femoral vein catheterization [4-5].

Since the original description by Aubaniac in 1952, SCV catheterization from the infraclavicular (IC) technique has developed into the most widely used and taught approach throughout the world [6]. In 1965, Yoffa introduced an alternate route to the SCV via the supraclavicular (SC) approach [7]. The purpose of the SC technique is to access the subclavian (SV) from its superior aspect just as it joins the internal jugular vein. The landmark for successful cannulation from this area is the clavisternomastoid angle formed by the muscle and clavicle. The needle is inserted at an avascular area lateral to the lateral head of the sternocleidomastoid muscle, directed at an angle of 45 degrees below the coronal plane aiming for the contralateral arm [7].

This route has some distinct advantages over the IC approach, including (i) more superficial access to the SV; (ii) a fixed, well-defined anatomical landmark; (iii) a bigger target area; (iv) a straight path for the guidewire and central venous catheters; and (v) less proximity to lung apices. In addition, SC access does not require interruption of cardiopulmonary resuscitation (CPR) or endotracheal intubation [8].

Thus, in the present study, we performed a systematic review to address the success rate and associated complications with the SC approach for SCV catheterization in adults for the identification of better practices and site selection in SCV access.

## Review

Methods

Search Strategy and Selection Criteria

**Figure 1:**
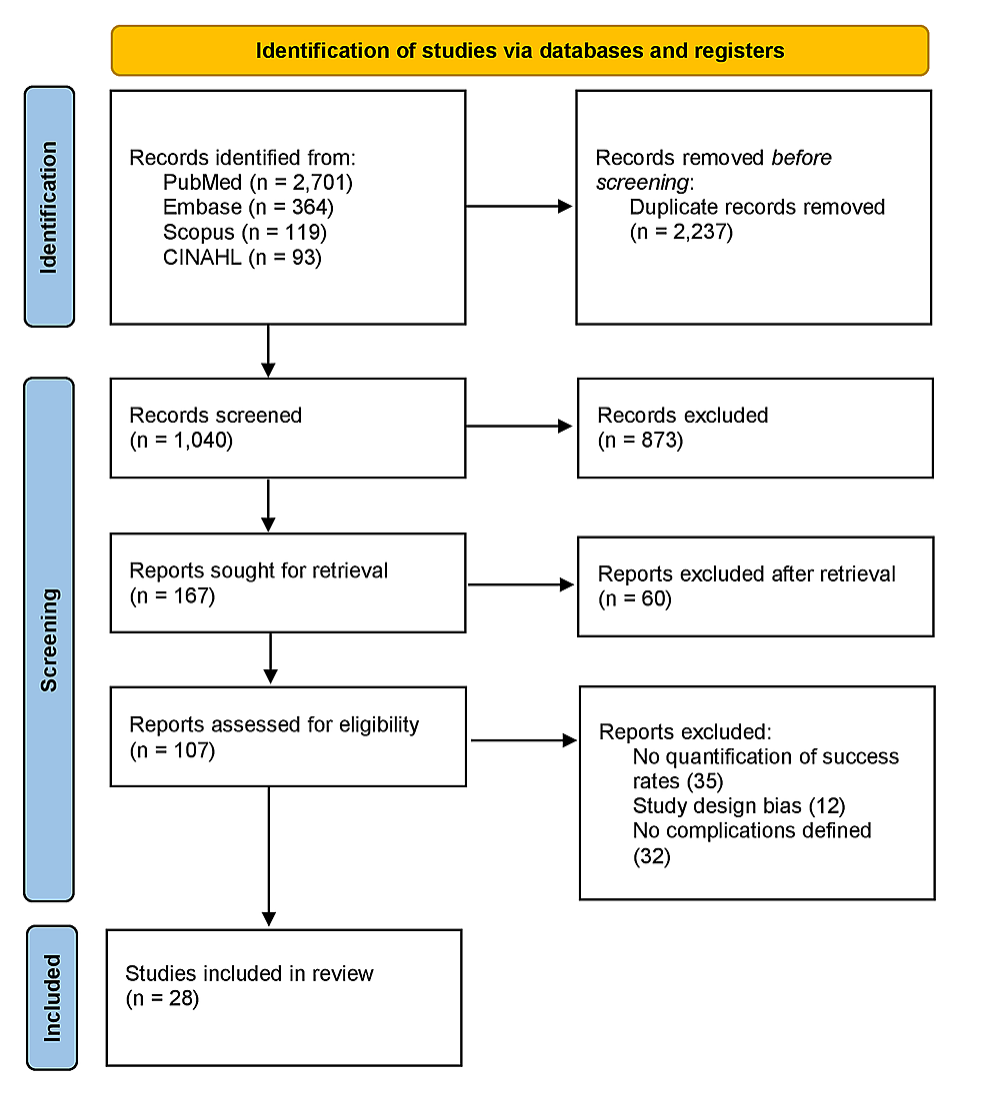
PRISMA flow chart PRISMA: Preferred Reporting Items for Systematic Reviews and Meta-Analyses

This systematic review adhered to the Preferred Reporting Items for Systematic Reviews and Meta-Analyses (PRISMA) guidelines [9]. Two reviewers (J.M. and M.K.) conducted a systematic, computerized search on the PubMed, Scopus, EMBASE, CINAHL, and Cochrane databases. In the search, we used MESH terms, including “supraclavicular approach” OR "supraclavicular technique” OR “subclavian vein catheterization” OR “central venous access,” and identified synonyms with controlled vocabularies. In addition, we checked the references of all included studies and relevant review articles identified by our search strategy. The search was last performed in January 2022 and English language studies, including case series, observational studies, and randomized controlled trials (RCTs) were deemed eligible for this review. Abstracts, editorials, study protocols, and letters to the editors were excluded. The decision was made with the consensus of the two authors (J.M. and M.K.). The opinion of the third author (S.M.J.Z.) was sought when an agreement could not be reached. The study selection process is presented in the PRISMA flow chart (Figure 1).

Outcome Measures

The overall cannulation success rate and any reported complication were regarded as the primary outcome measures while access time and incidence of malposition were taken as secondary outcomes.

Data Extraction

The following characteristics were extracted: name of the first author, publication year, males, study design, success rate, any reported complication, access time, and malposition. The data were extracted by two authors (J.M. and M.K.), and conflict was resolved through discussion and review.

Study Quality and Risk of Bias Assessment

The quality of each study was assessed by two authors (J.M. and M.K.) using the National Heart, Lung, and Blood Institute quality assessment tools, and studies were scored accordingly [10]. A score of 0-4 was labeled as poor, 4-7 as fair, and >7 as good quality studies. Study quality scores were not a key factor for exclusion.

Statistical Analysis

All data were analyzed using Review Manager 5.3 (Cochrane Collaboration, Oxford, UK.) and continuous variables were expressed as mean ± standard deviation (SD). Categorical variables (success rate, catheter malposition, and complications) were presented as frequency (n) and percentages (%).

Results

Study Selection and Characteristics

The literature searches from PubMed/MEDLINE, EMBASE, Scopus, and CINAHIL revealed a total of 3277 citations. Among these, 1040 were non-duplicated records. After the application of the inclusion and exclusion criteria, a total of 28 articles were included in the review. These 28 studies included a total of 14,790 patients. The baseline characteristics of the study participants along with study quality assessment are delineated in Table 1. The 28 studies published in peer-reviewed journals include six case series, eight randomized controlled trials, and 14 cohort studies (9 prospective and 5 retrospective). Studies were judged according to the National Heart, Lung, and Blood Institute quality assessment tool, and all were single-center studies.

Cannulation Success Rate

The overall success rate of the SC access ranged between 79% and 100%. Out of 28 studies, 24 studies had a success rate of above 90%. Many studies considered cannulation failure after three failed attempts of gaining central access. A total of 13 studies compared the success rate of the two techniques. Eleven studies demonstrated the SC technique to have more success in cannulating SCV compared with IC while two exhibited equal success rates (Table 1).

**Table 1:** Baseline study characteristics and outcomes * Study quality assessed by National Heart, Lung, and Blood Institute quality assessment tools. A score of 0-4 was labeled as poor, 4-7 as fair, and >7 as good quality studies. Not reported (NR); randomized controlled trial (RCT)

Author	Year	Study design	Controls	Sample size (for supraclavicular access); n	Success rate (Supraclavicular vs. Infraclavicular); %	Access time (min); mean ± Standard deviation	Overall complication rate; %	Malposition; n (%)	Pneumothorax; n (%)	Arterial puncture; n (%)	Hematoma; n (%)	Infection; n (%)	Thrombosis; n (%)	Obstruction; n (%)	Study quality*
Yoffa et al. [7]	1965	Case series	None	130	97.69	NR	0.76	NR	0 (0)	1 (0.76)	0 (0)	NR	NR	NR	6
Defalque et al. [11]	1970	Prospective cohort	None	1500	99	NR	0.46	NR	3 (0.20)	4 (0.26)	NR	NR	NR	NR	6
Haapaniemi et al. [12]	1974	Case series	None	600	93.66	NR	1.5	10 (1.66)	2 (0.33)	4 (0.66)	NR	3 (0.5)	NR	NR	7
Brahos et al. [13]	1977	Case series	None	100	96	NR	2	1 (1)	1 (1)	1 (1)	NR	NR	NR	NR	7
Brahos et al. [14]	1981	Case series	None	400	100	NR	0.75	3 (0.75)	2 (0.5)	1 (0.25)	NR	NR	NR	NR	6
Dronen et al. [15]	1982	Case series	Infraclavicular access	44	90.9 vs. 84.44	NR	2.27	3 (6.81)	0 (0)	1 (2.27)	NR	NR	NR	NR	5
Helmkamp et al. [16]	1985	Case series	None	99	90.9	NR	9	3 (3)	3 (3)	NR	NR	3 (3)	NR	3 (3)	4
Sterner et al. [17]	1986	RCT	Infraclavicular access	245	84.48	NR	1.62	1 (0.4)	2 (0.81)	2 (0.81)	NR	NR	NR	NR	6
Nessler e al. [18]	1987	Prospective cohort	None	9,042	97.3	NR	1.62	10 (0.11)	17 (0.18)	131 (1.44)	NR	NR	NR	NR	7
Jones et al. [19]	1992	Case series	None	34	79.41	NR	23.48	2 (5.88)	0 (0)	7 (20.58)	NR	1 (2.9)	0 (0)	0 (0)	6
Muhm et al. [20]	1997	Prospective cohort	None	208	94.71	NR	3.84	2 (0.96)	1 (0.48)	7 (3.36)	0 (0)	NR	NR	NR	6
Nevarre et al. [21]	1997	Prospective cohort	None	178	97.8	NR	0.56	1 (0.56)	1 (0.56)	NR	NR	NR	NR	NR	6
Apsner et al. [22]	1998	Prospective cohort	None	80	97.5	NR	8.6	3 (3.7)	1 (1.2)	3 (3.7)	NR	2 (2.5)	1 (1.2)	NR	5
Pittiruti et al. [23]	2000	Retrospective	Infraclavicular, internal jugular, external jugular, and femoral access	847	NR	NR	4.64	12 (1.4)	9 (1.1)	30 (3.54)	NR	NR	NR	NR	6
Czarnik et al. [24]	2009	Prospective cohort	None	370	88.9	NR	1.7	NR	0 (0)	3 (0.8)	0 (0)	NR	NR	NR	6
Pathiraja et al. [25]	2009	Prospective cohort	None	48	97.9	NR	4	1 (2.1)	1 (2.1)	0 (0)	0 (0)	1 (2.1)	NR	NR	4
Kocum et al. [26]	2011	RCT	Infraclavicular, jugular access	65	98 vs. 98	NR	0	4 (2.1)	0 (0)	0 (0)	0 (0)	NR	NR	NR	6
Hussain et al. [27]	2011	Prospective cohort	Infraclavicular access	72	95.83 vs. 87.5	NR	5.6	2 (2.8)	1 (1.4)	3 (4.2)	NR	NR	NR	NR	6
Aziz et al. [28]	2013	RCT	Infraclavicular access	69	92.8 vs. 78.3	NR	NR	NR	NR	NR	NR	NR	NR	NR	4
Thakur et al. [29]	2014	RCT	Infraclavicular access	30	96.99 vs. 90	4.3 ± 1.02	3.33	NR	0 (0)	1 (3.33)	NR	NR	NR	NR	4
Prasad et al. [30]	2015	Case series	None	50	88	4.8 ± 1.02	4	NR	0 (0)	2 (4)	NR	NR	NR	NR	6
Momin et al. [31]	2017	RCT	Infraclavicular access	25	92 vs. 80	5.3 ± 1.02	4	0 (0)	0 (0)	NR	1 (4)	NR	NR	NR	5
Tarbiat et al. [32]	2018	RCT	Infraclavicular access	140	94.3 vs. 97.1	NR	24.24	3 (2.14)	3 (2.14)	4 (2.8)	27 (19.3)	NR	NR	NR	5
Souadka et al. [33]	2020	Retrospective	Infraclavicular access	70	97.25 vs. 86.2	23 ± 8	2.84	NR	0 (0)	NR	1 (1.42)	1 (1.42)	0 (0)	0 (0)	6
Prasad et al. [34]	2020	c	Infraclavicular access	55	100 vs. 100	2.96 ± 0.20	NR	2 (3.63)	NR	NR	NR	NR	NR	NR	7
Javed et al. [8]	2020	Prospective cohort	Infraclavicular access	51	98 vs. 82.35	4.44 ± 1.07	3.92	NR	0 (0)	NR	2 (3.92)	0 (0)	0 (0)	NR	6
Khapung et al. [35]	2020	RCT	Infraclavicular access	35	100 vs. 88.57	1.9 ± 1.68	2.85	NR	0 (0)	0 (0)	1 (2.85)	NR	NR	NR	7
Kim et al. [36]	2021	RCT	Infraclavicular access	200	99.5 vs. 99.5	Median (Interquartile range): 9 (6–20)	2	2 (1)	0 (0)	4 (2)	0 (0)	NR	NR	NR	7

Access Time

**Figure 2:**
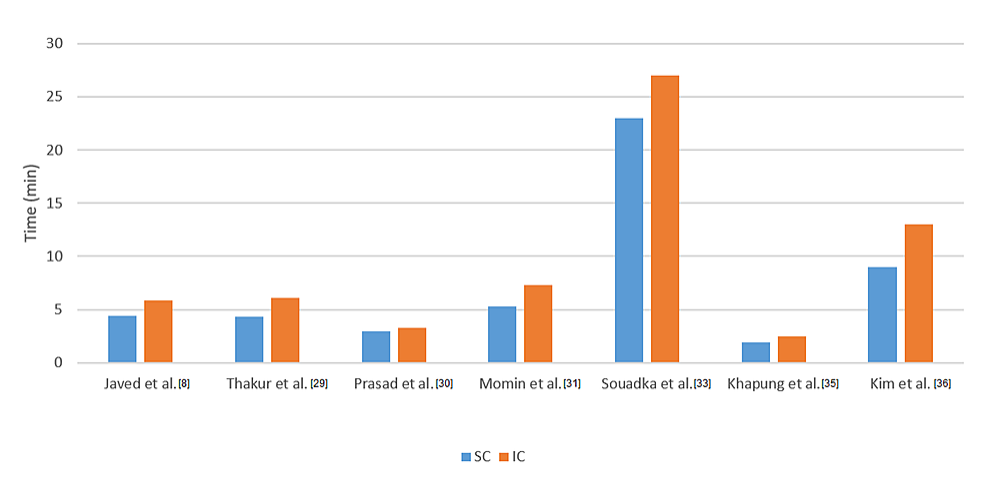
Comparison of access time between the supraclavicular and infraclavicular approaches

Five RCTs, one case series, and one prospective study investigated the time required for SVC cannulation using the two approaches. The time required for the SC approach ranged between 1.9 ± 1.68 and 23 ± 8 minutes. Seven studies compared access times between SC and IC catheterization and on average, the SC approach reduced catheterization time by 2.17 minutes. Access times are presented in Figure 2.

Discussion

**Figure 3:**
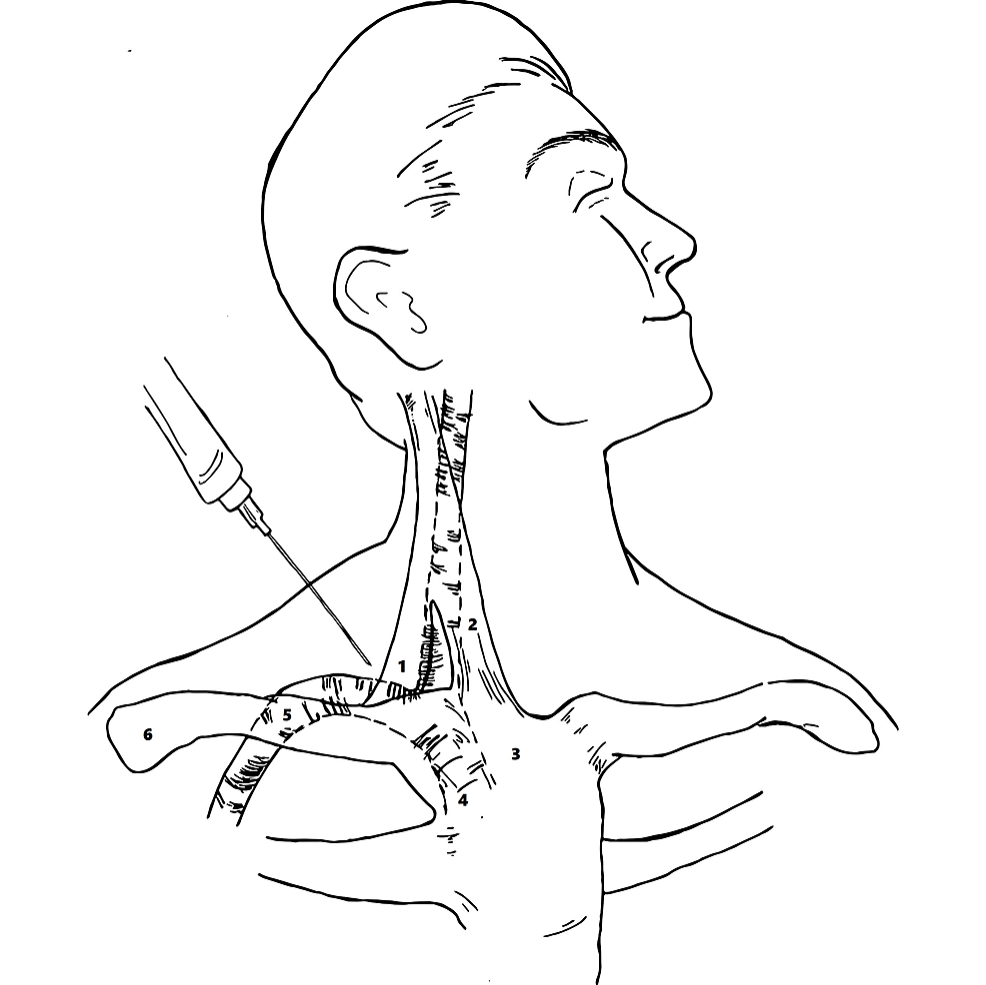
Surface anatomy of the neck indicating the procedure for inserting a central venous catheter into the subclavian vein via the supraclavicular approach The needle indicates the direction in which the access should be obtained. (1) Clavicular head of sternocleidomastoid, (2) sternal head of sternocleidomastoid, (3) manubrium, (4) right brachiocephalic vein, (5) right subclavian vein, (6) clavicle

The present study is the biggest qualitative synthesis (n=14,790) of the efficacy and safety of the SC approach for SCV in adults. After analyzing the results of 28 studies, the success rate for SC cannulation of SVC is high (mean of 98.17% vs. 89.33%) with a decreased access time (reduced time by 2.17 minutes) to cannulate SCV when compared with the IC approach [7-8,11-36]. Furthermore, there is a low incidence of associated complications and catheter malposition with SC access. This makes the SC approach of central venous access a feasible option for gaining rapid access in critical patients. Figure 3 shows the landmark technique for SC access.

Among all central venous catheterization, the preferred approach is the SCV due to easy localization, greater diameter, and high patient acceptance [3]. Moreover, SCV cannulation has a lower risk of associated catheter-related infection and thrombosis than an internal jugular or femoral vein cannulation [4-5]. There is a lot of literature on the two techniques of SCV access. However, in clinical practice, SC catheterization is not used as frequently as the IC approach because of the misconception of a higher risk to the patient in SCV access over the clavicle [37]. However, in this qualitative synthesis, we have observed that there was a low incidence of SC cannulation-associated complications.

As an anatomical site, the SC approach is more direct and accurate than the IC approach, hence, we demonstrated a decreased procedure time for the former technique. A study confirmed that SCV puncture through the SC technique is more superficial and has an easier insertion of guidewire than the IC technique, even with ultrasound guidance [30]. Furthermore, Kim and Prasad et al. have shown a better outcome in SC cannulation with ultrasound guidance and Czarnik et al. demonstrated no mechanical complications except arterial punctures [24,30,36]. This further confirms that cannulating SCV over the clavicle does not have an increased incidence of mechanical complications. Taken together, these observations suggest the use of the SC technique in critically ill patients where rapid venous access is required. Several studies indicate the use of the SC technique in various clinical settings. One study by Javed et al. shows that the SC approach has decreased cannulation time, fewer mechanical complications, catheter malposition, and overall fluoroscopy time in patients requiring a temporary pacemaker [8].

There are many uses for central venous catheter placement in critical care. Out of all these, volume resuscitation and invasive blood pressure monitoring are probably the most important in critical patients. Khapung et al. has demonstrated a comparison between the SC and IC approaches and the mean access time in group SC catheterization was 2.12 ± 0.81 min compared to 2.83 ± 0.99 min in group IC (p-value=0.002). Furthermore, the first attempt at central venous catheterization was successful in 74.28% of the patients as compared with 57.14% in the IC group [35]. Similarly, Souadka et al. exhibited that a long-term central venous access device for cancer chemotherapy can be performed more effectively with the SC approach [33]. The number of vein puncture attempts, the iatrogenic artery cannulation, and the operative time were more significant in the ICA group: (39,6 vs 17,6 p = 0,01), (9.2% vs 0; p = 0,01) and (27± 13 vs 23± 8min, p = 0.045), respectively. An RCT by Prasad et al. aimed to compare the access time and success rate of the SC approach with the IC technique under the guidance of ultrasonography. The total procedural time was significantly lower in the SC group than in the IC group (P < 0.0001). Time for localization, puncture, and catheterization was significantly higher in the IC group (P < 0.001). The success rate was 100% in both groups and the first attempt success rate was more in SC puncture. Similarly, an analysis of 370 mechanically ventilated patients by Czarnik et al. demonstrated that SCV via the SC approach is an excellent method of central venous access in such patients [24]. In 362 patients, vein localization attempts were performed, in whom 311 catheterizations (85.6%) were successful during the first attempt. The overall procedure complication rate reached 1.7% (95% confidence interval 0.6 - 3.6%). In a clinical investigation of 60 patients, a comparison of the SC with IC technique was undertaken, which demonstrated that the mean access time in the SC group was 4.30 ± 1.02 min compared to 6.07 ± 2.14 min in the IC group [29]. The overall success rate in catheterization of the right SCV using the SC approach was better as compared with the IC technique. First attempt success in the SC group was 75.6% as compared with 59.25% [29].

As for associated mechanical complications, this review demonstrated a comparable rate between the two SCV catheterization techniques (4.27%). One RCT by Kim et al. demonstrates the overall complication rate in SC and IC approach (3% vs. 13.4%, mean difference (95%CI) -10.4% (-15.7 to -5.1%). P < 0.001). In addition, there was a significant difference for catheter malposition and time required for venous puncture (median (IQR (range)): 9 (6-20 (2-138)) vs. 13 (8-20(3-99))) [36]. Similarly, Pittiruti et al. showed a low incidence of pneumothorax (1.1% vs. 2.5%, p < 0.001), repeat procedure (4% vs. 6.5%, p < 0.001), and malposition (1.4% vs. 2.6%, p < 0.001) [23]. Souadka et al., in addition to the low incidence of mechanical complications, describe a low patient pain perspective probably due to the proximity of the pleura and lung cavity, as well as the distance from the skin to the vein [33]. Thakur et al. demonstrated an increased complication rate in the SC compared with the IC technique [29]. In a study by Tarbiat et al., three catheters were misplaced in the SC group and seven in the IC group, three patients had pneumothorax with the SC approach and one had pneumothorax with the IC technique, and hematoma at the puncture site occurred in one patient with the IC approach and 27 patients with the SC approach. In contrast, the majority of studies demonstrated low complication rates in the SC technique [24,29,31,33].

Limitations

Our systematic review had several limitations to note. First, inclusion criteria only addressed studies carried out on adult patients. Second, only two studies examined ultrasound-guided SCV puncture. Further studies and randomized trials are needed to clarify the effectiveness of this imaging modality on the success and complication rates of both techniques of SCV puncture. Third, quantitative synthesis was not carried out due to the observational nature of the majority of the included investigations. The RCTs were not double-blind because they compared different techniques. Ultrasound guidance was not an inclusion criterion for the selected studies because at our institute landmark technique is used in all cases as ultrasound guidance is not available.

## Conclusions

In conclusion, this systematic review shows that the SC technique is a practical alternative to the IC approach in terms of overall success rate and access time, with no increase in the rate of mechanical complications. We found fewer catheter-related mechanical complications, including fewer catheter malposition and faster venous puncture. These findings indicate that the SC technique can be utilized in rapid venous access in critically ill patients, pacemaker insertion, and extracorporeal membrane oxygenation. Further studies on the role of ultrasound guidance are warranted for the SC approach.
